# Macroalgal Defense against Competitors and Herbivores

**DOI:** 10.3390/ijms22157865

**Published:** 2021-07-23

**Authors:** Gracjana Budzałek, Sylwia Śliwińska-Wilczewska, Kinga Wiśniewska, Agnieszka Wochna, Iwona Bubak, Adam Latała, Józef Maria Wiktor

**Affiliations:** 1 Division of Marine Ecosystems Functioning, Institute of Oceanography, University of Gdańsk, P-81-378 Gdynia, Poland; gbudzalek@gmail.com (G.B.); oceal@ug.edu.pl (A.L.); 2 Division of Marine Chemistry and Environmental Protection, Institute of Oceanography, University of Gdańsk, P-81-378 Gdynia, Poland; kinga.wisniewska@phdstud.ug.edu.pl; 3 GIS Centre, Institute of Oceanography, University of Gdańsk, P-81-378 Gdynia, Poland; agnieszka.wochna@ug.edu.pl; 4 Division of Hydrology, Institute of Geography, University of Gdansk, P-80-309 Gdańsk, Poland; iwona.bubak@ug.edu.pl; 5 Department of Marine Ecology, Institute of Oceanology of the Polish Academy of Sciences, P-81-779 Sopot, Poland; wiktor@iopan.gda.pl

**Keywords:** aquatic animals, allelopathy, allelochemicals, chemical defense, defense strategy, plant defense, species interactions

## Abstract

Macroalgae are the source of many harmful allelopathic compounds, which are synthesized as a defense strategy against competitors and herbivores. Therefore, it can be predicted that certain species reduce aquaculture performance. Herein, the allelopathic ability of 123 different taxa of green, red, and brown algae have been summarized based on literature reports. Research on macroalgae and their allelopathic effects on other animal organisms was conducted primarily in Australia, Mexico, and the United States. Nevertheless, there are also several scientific reports in this field from South America and Asia; the study areas in the latter continents coincide with areas where aquaculture is highly developed and widely practiced. Therefore, the allelopathic activity of macroalgae on coexisting animals is an issue that is worth careful investigation. In this work, we characterize the distribution of allelopathic macroalgae and compare them with aquaculture locations, describe the methods for the study of macroalgal allelopathy, present the taxonomic position of allelopathic macroalgae and their impact on coexisting aquatic competitors (Cnidaria) and herbivores (Annelida, Echinodermata, Arthropoda, Mollusca, and Chordata), and compile information on allelopathic compounds produced by different macroalgae species. This work gathers the current knowledge on the phenomenon of macroalgal allelopathy and their allelochemicals affecting aquatic animal (competitors and predators) worldwide and it provides future research directions for this topic.

## 1. Introduction

Aquaculture has rapidly grown over the past few decades and is now the fastest-growing food sector worldwide [1]. The global aquaculture production in 2015 was approximately 106 million tons, which represents approximately 163 billion US dollars [2]. The global population has been increasing and is expected to reach ~10 billion in the middle of the 21st century [3]. The corresponding increase in food demand is driving the expansion of aquaculture [4]. The pressure on these food sectors to maximize production and reduce losses is also expected to increase [2].

A popular method to increase aquaculture production is to enrich farming tanks with macroalgae species. Macroalgae as a food source believed to be an ideal candidate for growth in fishponds because they provide high biomass production and protein content [5]. Additionally, the environment of the ponds is improved by macroalgae through the balance of pH levels [6]. Different macroalgal species have been integrated into land-based integrated multi-trophic aquacultures (IMTA) for biomass production [7]. The high amount of protein from macroalgae represents valuable feed for animal species with high commercial value [5,7]. However, studies on this topic rarely mention that allelopathic macroalgae can negatively affect and even exterminate both competitors and predators by secreting a broad range of harmful and toxic substances such as acetogenins, alkaloids, aromatic compounds, fluorotannins, polyphenols, terpenes, and amino acids [8].

Macroalgal allelopathy refers to the effects of substances produced by the microalgae on target organisms [9]. These effects can be related to the growth, health, origin, or population biology of the donor and target organisms [8,9]. The allelopathic activity of macroalgae is a complex process. It is considered that its level depends on the production of active allelopathic compounds and their effective escalation to accompanying organisms [10]. Macroalgae are mainly benthic organisms firmly attached to the seabed, which forces them to compete for substrates, nutrients, and light with other benthic organisms. There are also unattached forms of macroalgae [11], which can influence the development of planktonic organisms. Kersen [11] showed that the unattached forms of *Furcellaria lumbricalis* and *Coccotylus truncatus* can be considerably denser than their respective attached forms. Therefore, their deleterious effects on other organisms can be stronger than those of benthic algae. Nevertheless, their allelopathic activities have not been sufficiently investigated.

**Figure 1:**
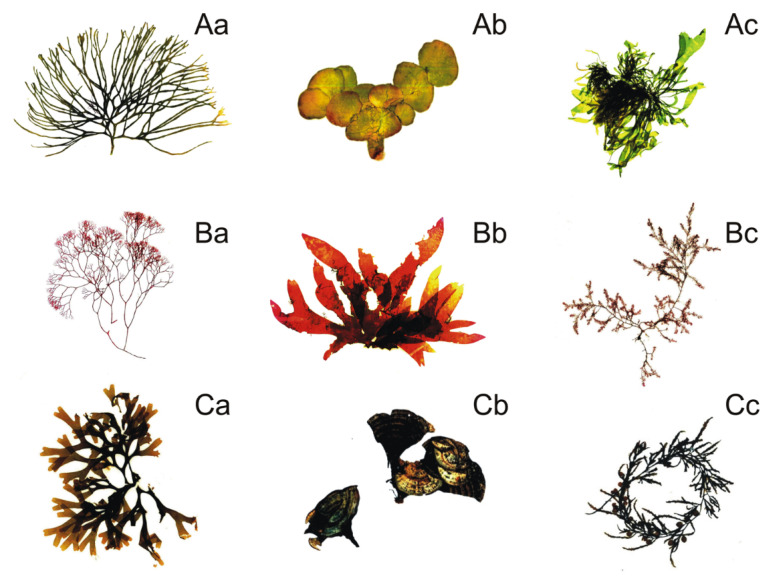
Examples of allelopathic green algae (**A**): *Codium fragile* (**a**), *Halimeda tuna* (**b**), *Ulva* sp. (**c**); red algae. (**B**): *Ceramium rubrum* (**a**), *Grateloupia* sp. (**b**), *Polysiphonia* sp. (**c**); brown algae. (**C**): *Dictyota* sp. (**a**), *Padina* sp. (**b**), *Sargassum* sp. (**c**).

Studies related to the impact of macroalgae on other organisms have mainly focused on marine environments [8,12,13]. However, freshwater and brackish macroalgae can also achieve rapid biomass increase, which can result in algal blooms [14,15,16]. Moreover, macroalgae from freshwater and brackish ecosystems can negatively affect the growth of photoautotrophs [17,18]. Nevertheless, there is little research on the impact of these organisms on coexisting aquatic animals. Macroalgae in marine environments belong to three groups: Ulvophyceae, Chlorophyta (green algae), Florideophyceae, Rhodophyta (red algae), and Phaeophyceae, Ochrophyta (formerly Phaeophyta; brown algae), whereas those from freshwater include mainly Ulvophyceae, Chlorophyta and Charophyceae, Charophyta [19]. Macroalgae with confirmed allelopathic activity against other heterotrophic organisms are shown in Figure 1.

Recently, research on the allelopathy phenomenon has increased significantly [8,13,20]; however, to the best of our knowledge, no published review has revealed the negative effects of macroalgae on coexisting competitors and predators. In this work, we (i) characterize the distribution of allelopathic macroalgae and compare them with aquaculture locations, (ii) describe the methods for the study of macroalgal allelopathy, (iii) present the taxonomic position of allelopathic macroalgae and their impact on coexisting animal competitors (Cnidaria species) and herbivores (Annelida, Echinodermata, Arthropoda, Mollusca, and Chordata species), and (iv) compile information on allelopathic compounds produced by different macroalgae species. This work gathers the current knowledge on the phenomenon of macroalgal allelochemicals affecting aquatic competitors and herbivores worldwide and it provides future research directions for this topic.

## 2. Distribution of Allelopathic Macroalgae and Aquaculture Locations

In this work, the allelopathic effect of green algae (Chlorophyta, Ulvophyceae), red algae (Rhodophyta, Florideophyceae), and brown algae (Ochrophyta, Phaeophyceae) was investigated against different aquatic animals. Allelopathic activity has been reported for a total of 123 taxa, including 37 green algae (30%), 45 red algae (37%), and 41 brown algae (33%). The allelopathic ability of 11 different genera of Chlorophyta, 28 genera of Rhodophyta, and 13 genera of Ochrophyta has been reported (Figure S1, Table S1). The allelopathic activity of macroalgae has most often been studied in Chlorophyta from the genera *Caulerpa*, *Chlorodesmis*, and *Ulva*. *Hypnea* sp. has been the most frequently studied among Rhodophyta for allelopathy. Among the allelopathic Ochrophyta, *Dictyota* sp. and *Lobophora* sp. have been the most frequently studied. The least numerous studies for allelopathic ability have been conducted for organisms belonging to *Anadyomene*, *Codium*, *Penicillus*, and *Rhiphilia* (green algae); *Asparagopsis*, *Callophycus*, *Centroceras*, *Ceramium*, *Chondria*, *Chondriopsis*, *Chondrophycus*, *Crassiphycus*, *Delisea*, *Dermonema*, *Digenea*, *Endosiphonia*, *Peyssonnelia*, *Phacelocarpus*, *Plocamium*, *Polysiphonia*, *Tayloriella*, *Tichocarpus*, and *Yuzurua* (red algae); and *Canistrocarpus*, *Desmarestia*, *Dictyopteris*, *Dilophus*, *Ecklonia*, *Laminaria*, and *Sphacelaria* sp. (brown algae).

**Figure 2:**
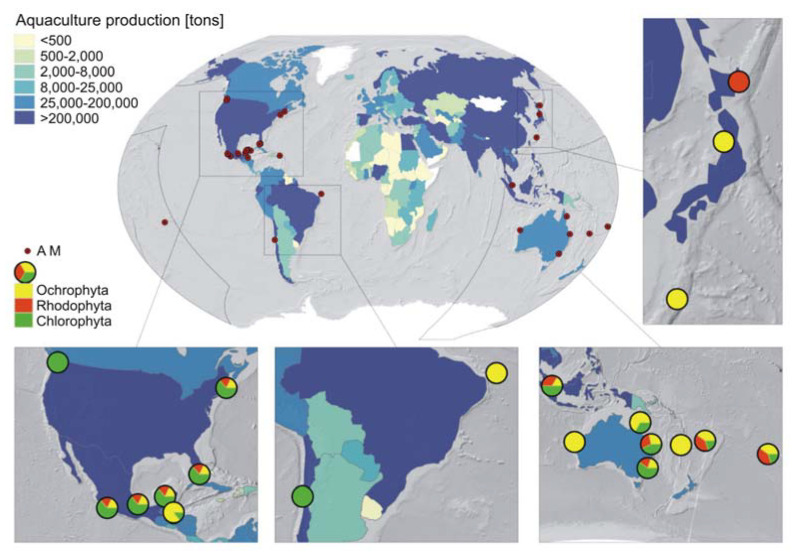
Allelopathic macroalgae (AM) in the studied areas based on the donor species found in the literature compared to the places where world aquaculture production occurs (based on the World Bank data; https://data.worldbank.org/indicator/ER.FSH.AQUA.MT, accessed on 17 June 2021).

Research on macroalgae and their allelopathic effects on other organisms has been primarily conducted in Australia, Mexico, and the United States (Figure 2). Nevertheless, a few scientific investigations have been conducted in South America and Asia in areas coinciding with aquaculture activity (Figure 2). In most areas, all three phyla were tested. However, the studies in some regions focused only on one macroalgae phylum. *Chlorodesmis fastigiata* is the most studied green algae, accounting for 30.4% of all tested organisms of this phylum [21,22,23,24,25,26]. In studies on brown algae, *Dictyota bartayresiana* dominates, accounting for 12.5% of the total studies [22,24,27], whereas in red algae, *Galaxaura filamentosa* is the most widely investigated, with studies accounting for 13.6% [22,23,24].

## 3. Methods for Macroalgal Allelopathy Examination

**Figure 3:**
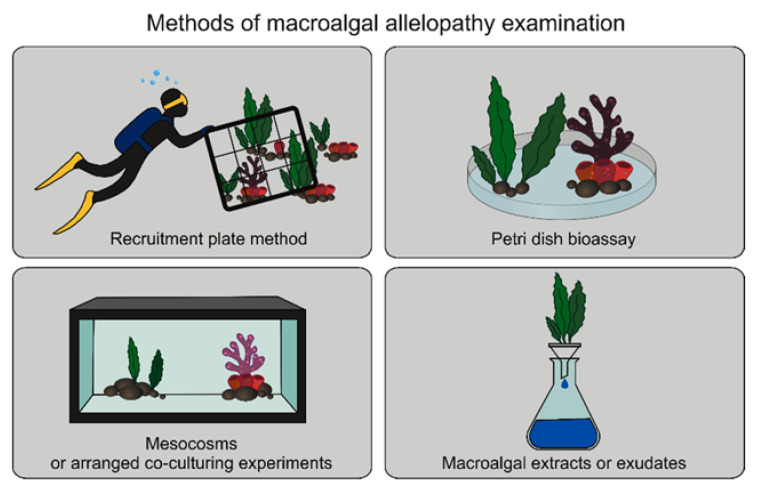
Most used methods to investigate the allelopathy phenomenon.

To recognize the allelopathy impact of macroalgae on coexisting aquatic animals (competitors and herbivores), many investigation methods are necessary, from field observation to co-culturing experiments in mesocosms. Most studies on the allelopathic activity of macroalgae on target aquatic animals are characterized by a specific method suited to test those organisms and environment. Four main methods for testing macroalgal allelopathy are shown in Figure 3. In the most used method, the recruitment plate method, the impact of macroalgae on animals is examined by observing the settlement degree of target organisms and their survival rate on specially arranged tiles placed in the field [21,23,28,29]. In the second most-used method, the effect of macroalgal extracts or exudates on the development and survival of target animals is analyzed [8,30,31,32,33,34,35,36,37,38,39]. The third method includes the analysis of the interaction of macroalgae or their compounds on animals tested in a petri dish [40,41]. Finally, experiments in mesocosms or arranged co-culturing experiments for algae and animals are conducted [25,27,42].

## 4. Taxonomic Position of Allelopathic Macroalgae and Their Impact on Coexisting Competitors and Herbivores

Macroalgae are major competitors for the light and space for corals and other benthic organisms from the Cnidaria phylum on tropical reefs [43]. Competition can occur through direct and indirect physical and chemical mechanisms reviewed in detail by Chadwick and Morrow [44]. Macroalgae can produce inhibitory compounds affecting corals and epibionts that compete for light or space [9]. Globally, many coral reefs have been damaged, and areas with reduced coral cover and increased macroalgal abundance have been widely identified [45]. Despite the well-documented negative correlation between macroalgae and coral recruitment, the mechanisms through which macroalgae affect this recruitment have received little attention.

In addition, macroalgal allelopathy has an important and as-yet unrecognized role in structuring temperate shallow marine communities of herbivores: Annelida (e.g., *Sabellaria cementarium* and *Spinoidae* sp.) [41], Echinodermata (e.g., *Holopneustes purpurascens*, *Lytechinus variegates*, and *Strongylocentrotus intermedius*) [31,33,35,36], and Arthropoda species (*Cancer oregonensis*, *Metacarcinus magister*, and *Pachygrapsus transversus*) [35,46]. Furthermore, several researchers have reported the negative effects of macroalgae on Mollusca species e.g., [38,47,48]; they suggested that green macroalgae species (especially from the Ulvophyceae class) can inhibit the growth and development of co-occurring organisms from the genus *Crassostrea*. Moreover, oyster larvae (e.g., *Crassostrea gigas*) are susceptible to extracts from *Ulvaria lactuca* thallus at relatively low concentrations [48]. Although several researchers have reported both negative and positive effects of green algae species on invertebrates [41,46,49,50], few studies have reported the potential effects of *Ulva* sp. on the economically relevant Mollusca, *Crassostrea virginica* [38]. Many aquaculture farms cultivate *C. virginica* in areas where *Ulva* is present. Research has also shown that macroalgae can adversely affect species belonging to the Chordata phylum [8,30,31,32]. Moreover, certain investigated fishes that belong to *Carassius* sp. and *Tilapia* sp. are consumed by humans. As contribution of aquatic animals to global food is crucial, such results are alarming and warrant special attention [2].

**Figure 4:**
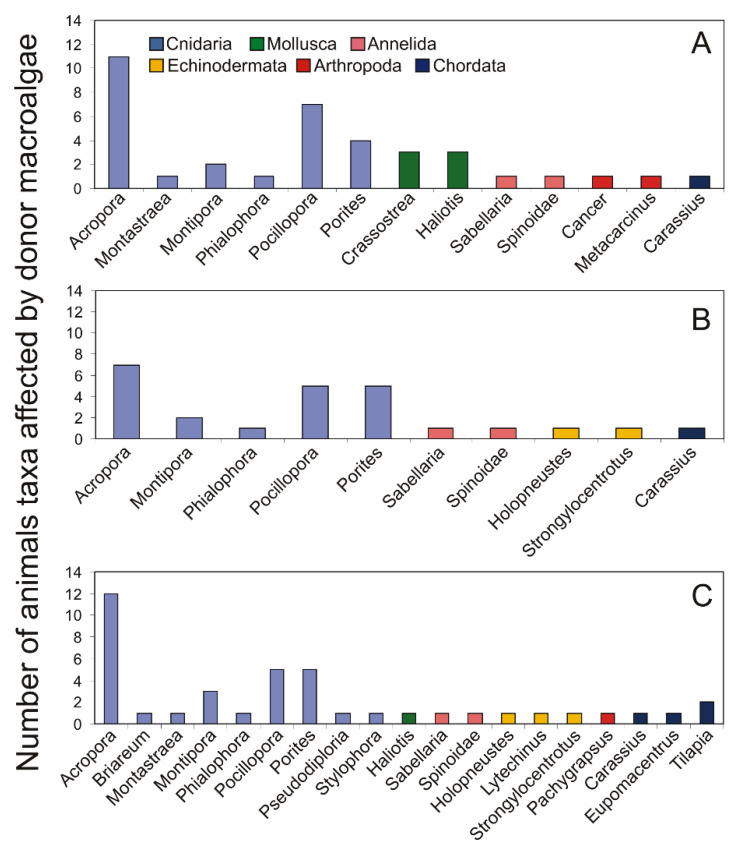
Number of target competitors and herbivores affected by green algae (**A**), red algae (**B**), and brown algae (**C**), based on taxa found in the literature.

The interactions of green algae on 13 different genera of aquatic animals (both competitors and predators) have also been reported (Figure 4). The allelopathic activity of Chlorophyta species was tested against six taxa belonging to Cnidaria, two to Mollusca, two to Annelida, two to Arthropoda, and one to Chordata phylum. Conversely, the influence of red algae was investigated on ten aquatic animals (five belonging to Cnidaria, two to Annellida, two to Echinodermata, and one to Chordata). Overall, the greatest number of animal species have been tested for their sensitivity to brown algae. The allelopathic activity of these macroalgae was tested against 19 genera of different aquatic animals. Allelopathic activity of brown algae was tested on animals belonging to the Cnidaria, Mollusca, Annelida, Echinodermata, Arthropoda, and Chordata phyla. As in the case of other macroalgae, the allelopathic activity of brown algae has been most frequently studied for taxa belonging to the Cnidaria. Animals belonging to the genus *Crassostrea* and *Haliotis* (Mollusca), *Strongylocentrotus* (Echinodermata), *Cancer* and *Metacarcinus* (Arthropoda) as well as *Carassius* and *Tilapia* (Chordata), are commonly used in aquaculture. Therefore, it is important to further investigate and compare information on the interactions between macroalgal species and economically important animals.

### 4.1. The Allelopathic Activity of Green Algae

The allelopathic activity of green algae (Ulvophyceae, Chlorophyta) was confirmed by several authors (Table 1). Studies have shown that the presence of green algae has a generally negative effect on Cnidaria [21,22,23,24,25,26,28,39,51,52]. Tanner [21] was the first author who showed that *Chlorodesmis fastigiata* and *Halimeda* sp. had a negative impact on *Acropora* (*Isopora*) *cuneata*, *Acropora hrueggemanni*, *Acropora palifera*, and *Pocillopora damicornis*. Similar research was conducted by Rasher et al. [22]. Andras et al. [51] proved that the green alga *Rhiphilia pencilloides* caused coral bleaching when placed in contact with *Porites rus*. Morrow et al. [52] showed the impact of macroalgal extracts obtained from *Halimeda tuna* on the sublethal stress response of corals. In turn, Bonaldo and Hay [23] investigated macroalgae-coral interactions considering both non-allelopathic and allelopathic species. Furthermore, Lee et al. [28] examined the effects of macroalgal species on the settlement success of *P. damicomis* larvae under aquarium conditions. Ritson-Williams et al. [24] examined that *C. fastigiata* negatively affects *A. millepora*, *M. digitata*, and *P. damicornis*. Fong et al. [39] showed that the mortality of *Pocillopora acuta* larvae increased significantly with an increase in the concentration of the crude extract obtained from *Bryopsis* sp. Longo and Hay [26] demonstrated that the lipid-soluble extracts obtained from the green alga *C. fastigiata* suppressed coral *Pocillopora verrucosa* photochemical efficiency. Conversely, Del Monaco et al. [25] showed that donor macroalgae *C. fastigiata* damages corals via allelopathy regardless of CO_2_ concentration. Only Birrell et al. [40] described a positive and neutral effect of Chlorophyta on Cnidaria. These authors demonstrated that *C. fastigiata* caused a slight delay in the settlement of coral larvae; however, these results were not statistically significant. Green-Gavrielidis et al. [38], Nelson et al. [47], and Nelson and Greg [48] have shown that macroalgae from the genus *Ulva* have had a negative impact on Mollusca. Green-Gavrielidis et al. [38] showed that bloom-forming *Ulva compressa* negatively affected the growth of *Crassostrea virginica* and the strongest effect was seen in larvae exposed to *U. compressa* exudates growing on nutrient-sufficient medium. Nelson et al. [47] and Nelson and Greg [48] showed that oyster larvae (*Crassostrea gigas*) are susceptible to extracts from dried *Ulva lactuca* and *Ulvaria obscura* at relatively low concentrations. Conversely, Muñoz et al. [50] showed that the presence of *Ulva* sp. improved the growth rate of the *Haliotis rufescens* larvae, while Huggett et al. [49] noted high colonization of *Haliotis rubra* in the presence of *Ulva australis*, *Ulva compressa*, and *Ulvaria obscura*. Warkus et al. [41] were the only authors who studied the influence of Ulvophyceae on Annelida (Table 1). This work demonstrated the negative effect of *Chaetomorpha* sp., *Codium fragile*, *Ulva* sp. (formerly *Enteromorpha* sp.), and *Ulva lactuca* on polychaeta *Sabellaria cementarium* and *Spinoidae* sp. In turn, the diverse effects of *Ulvaria obscura* on Arthropoda have been described by Van Alstyne et al. [46]. The authors demonstrated that tested green algae did not affect the survival of *Cancer oregonensis* and *Metacarcinus magister* juveniles. It was also shown that *U. obscura* had little effect on the time of first molting of these animals. Alvarez-Hernández et al. [8] showed that various species belonging to Chlorophyta were considered highly toxic to Chordata (the goldfish *Carassius auratus auratus*) when acetonic or ethanolic extract was made. The most toxic Chlorophyta were: *Caulerpa cupressoides*, *Caulerpa racemosa*, *Chaetomorpha antennina*, and *Penicillus capitatus*. However, aqueous extract obtained from these green algae had no effect on *C. auratus auratus* (Table 1).

Many macroalgae, such as *Ulva* sp., are cosmopolitan organisms, and in nutrient-rich coastal waters, they are often dominant and bloom-forming species [15,53,54]. These studies confirm that Chlorophyta may have a negative impact on co-occurring animal organisms. Therefore, allelopathy phenomenon of species belonging to Chlorophyta on coexisting animal organisms should be widely studied in the future.

### 4.2. The Allelopathic Activity of Red Algae

The allelopathic activity of red algae (Florideophyceae, Rhodophyta) on coexisting animals has also been confirmed by a few experimental studies (Table 2). The negative effect of red algae on Cnidaria was described by Tanner [21], Rasher et al. [22], Bonaldo and Hay [23], Ritson-Williams et al. [24], Del Monaco et al. [25], Longo and Hay [26], Fong et al. [39], and Andras et al. [51]. In addition, a few authors [22,24,39,42] observed that certain red algae species had no allelopathic effect on target Cnidaria (Table 2). Tanner [21] described that *Acropora* species growing faster in areas from which red macroalgae *Peyssonnelia* sp. had been removed compared to control areas where Rhodophyta species were present. Similarly, Andras et al. [51] used field experiments to show that contact with the red algae *Callophycus densus*, *Phacelocarpus neurymenioides*, and *Plocamium pacificum* induces bleaching on natural colonies of *Porites rus*. Moreover, the corals in the control experiments, in which they encountered plastic imitation algae, showed no bleaching, which may suggest the effect of the red macroalgae allelochemicals rather than the effect of shading or physical contact. Bonaldo and Hay, [23] demonstrated that the presence of allelopathic red macroalgae *Galaxaura filamentosa* caused faster and more extensive damage to *Acropora aspera* and *P. damicornis* than to *Porites cylindrica*, *Porites lobata*, and *Montipora digitata*. Furthermore, Longo and Hay [26] showed that the red algae *Amansia rhodantha* and *Asparagopsis taxiformis* extracts negatively affected the photochemical efficiency of the coral *Phialophora verrucosa*. Fong et al. [39] examined the effects of crude extracts from macroalgal species *Endosiphonia horrida* and *Hypnea pannosa* on *Pocillopora acuta* larvae. In turn, Del Monaco et al. [25] showed that common Rhodophyta *Amansia glomerata* damage corals *Acropora intermedia* via allelopathy, however, the effect of the macroalgal extracts was not stronger when the tested Rhodophyta species were grown under elevated CO_2_ conditions. Rasher et al. [22] and Ritson-Williams et al. [24] showed that red algae *G. filamentosa* had negative effects on *Acropora millepora*, *M. digitate*, and *P. damicornis*. Similarly, Kuffner et al. [42] demonstrated no allelopathic effects of *Chondrophycus poiteaui* (formerly *Laurencia poiteaui*) on the recruitment success of *Porites astreoides* larvae. Moreover, Warkus et al. [41] described the negative influence of Rhodophyta *Grateloupia turu turu* and *Polysiphonia denudata* on Annelida *Sabellaria cementarium* and *Spinoidae* sp. Ishii et al. [36] also demonstrated that compounds obtained from red algae (*Tichocarpus crinitus*) exhibited feeding-deterrent properties against the Echinodermata *Strongylocentrotus intermedius*. Conversely, Williamson et al. [33] showed that allelochemicals produced by *Delisea pulchra* caused a positive effect on metamorphosis and triggered settlement in other Echinodermeta *Holopneustes purpurascens*. The studies by Alvarez-Hernández et al. [8] showed that, in general, the aqueous extract did not affect the behavior of the *Carassius auratus auratus* belonging to Chordata phylum. The only exception was *Chondriopsis dasyphylla* f. *pyrifera*, which showed strong toxicity to the tested animal after exposure to aqueous, acetonic, and ethanolic extracts. The studies by Alvarez-Hernández et al. [8] showed that the activity of macroalgae also depends on the place of occurrence of individual species.

### 4.3. The Allelopathic Activity of Brown Algae

Ochrophyta (Phaeophyceae) were the most frequently studied organisms among all macroalgal phyla in which allelopathic activity on target organisms was confirmed (Table 3). The strong negative impact of brown algae on Cnidaria has been described in detail by Tanner [21], Del Monaco et al. [25], Webster et al. [29], Fong et al. [39], Kuffner et al. [42], Paul et al. [55], and Olsen et al. [56]. Tanner [21] demonstrated that changes in *Acropora* sp. cover were significantly affected by the presence of this brown algae. Later, Kuffner et al. [42] showed that tested brown algae (*Dictyota menstrualis* and *Lobophora variegata*) inhibited recruitment and avoidance behavior in *Porites astreoides* larvae. Olsen et al. [56] also provided evidence that the presence of the brown alga *D. menstrualis* has direct negative effects on the survival and recruitment of Caribbean coral *P. astreoides*. Moreover, Webster et al. [29] showed the negative effect of brown algae *Sphacelaria* sp. on larval settlement and the growth as well as the survival of coral recruits *Acropora millepora*. Fong et al. [39] demonstrated that mortality of *Pocillopora acuta* larvae increased considerably with increasing concentrations of *Lobophora* sp. extracts. Furthermore, Del Monaco et al. [25] shown that elevated CO_2_ concentrations increased the deleterious effect of *Canistrocarpus* (=*Dictyota*) *cervicornis* on *Acropora intermedia*. In turn, Paul et al. [55] provided evidence that *Dictyota pulchella* and *Dictyota pinnatifida* may adversely affect larval settlements and recruitment.

Several studies have shown that brown algae can have different effects on animals depending on the donor and target species [22,24,26,27,28,52,57]. Lee et al. [28] examined the effects of macroalgal species on the settlement success of *Pocillopora damicomis* larvae under aquarium conditions. Longo and Hay [26] also conducted field experiments assessing the effects of extracts obtained from *Dictyota bartayresiana* and *Turbinaria ornata* on the coral *Pocillopora vcerrucosa*. Ritson-Williams et al. [24] showed that the brown algae *D. bartayresiana* negatively affected *Acropora millepora*, *Montipora digitata*, and *P. damicornis*. Four years later, Ritson-Williams et al. [27] tested settlements in the presence of different algae of three coral species: *Acropora palmata*, *Acropora cervicornis*, and *Pseudodiploria strigosa*. Vieira et al. [57] showed that extracts obtained from *Lobophora* sp. can bleach certain coral species during direct contact. Furthermore, the authors demonstrated that the studied corals differed in their sensitivity to the presence of an extract obtained from brown algae. In turn, Morrow et al. [52] found that both the crude extracts and the presence of live brown algae induced significant changes in the bacterial complex associated with corals and sublethal stress responses in *Montastraea faveolata*. Furthermore, Rasher et al. [22] demonstrated that macroalgae can directly cause bleaching and death of corals by the transfer of hydrophobic allelochemicals present on their surfaces. It was found that damage to corals has generally been confined to places where it encounters the macroalgae. However, contact with the corals had no effect on these brown algae species. These findings suggest that the deleterious effects on corals are caused by allelopathic compounds rather than by physical contact. Conversely, Birrell et al. [40] have shown that Ochrophyta (*Lobophora variegata*) can also have a positive effect on Cnidaria *Acropora millepora*. To study allelopathic compounds that control seaweed-herbivore interactions, Suzuki et al. [34] investigated the effects of *Dilophus okamurae* on Mollusca (*Haliotis discus hannai*). Only Warkus et al. [41] described the negative allelopathic effect of brown algae *Desmarestia viridis* and *Laminaria* sp. on polychaeta *Sabellaria cementarium* and *Spinoidae* sp. (Annelida). Barbosa et al. [35] showed that compounds obtained from *Dictyota pfaffii* were effective in inhibiting feeding by the sea urchin *Lytechinus variegatus* (Echinodermata). Research conducted by Gerwick and Fenical [31] also confirmed the negative effect of Ochrophyta on Echinodermata. Conversely, Williamson et al. [33] showed that *Ecklonia radiata* had no effect on the development and metamorphosis of *Holopneustes purpurascens* (Echinodermata) larvae. Barbosa et al. [35] were the only authors who documented that the compound obtained from *Dictyota pfaffii* did not inhibit feeding by the crab *Pachygrapsus transversus* (Arthropoda). Research conducted by Alvarez-Hernández et al. [8] showed that brown algae may adversely affect animals belonging to Chordata phylum. Gerwick et al. [30] performed an experiment showing that when *Stypopodium zonale* was placed in the aquarium, the water became a rust colored and toxic to the herbivorous fish *Eupomcentrus leucostictus.* Later, Gerwick and Fenical [31] described that nearly all the compounds isolated from *S. zonale* showed negative effects on the same species of reef-dwelling fish. It has been suggested that the production of noxious and allelopathic substances contributes significantly to the survival of *S. zonale* in predator-rich areas in which it abounds.

All these results indicate that brown algae may affect the marine ecosystem by limiting the development of associated animals. Moreover, recent field assays have suggested the potential role of chemical mediators in this interaction. It has also been suggested that certain brown algae species may produce allelopathic compounds that may play an important ecological function as a defense strategy against herbivores worldwide [35].

## 5. Allelopathic Compounds Produced by Macroalgae

Since there is very little information about the compounds produced by macroalgae, this section provides examples of characterized macroalgae compounds that interact with other heterotrophic organisms (not only competing and herbivorous).

Many studies have reported novel secondary metabolites produced by marine Chlorophyta species, which have significant biological activity on target organisms (Table 4). Capisterones, caulerpals, cycloeudesmol, cymobarbatol, halitunal, isorawsonol, lyengaroside, and sphingosin are compounds that have been isolated from *Penicillus capitatus*, *Caulerpa taxifolia*, *Chondria oppositiclada*, *Cymopolia barbat*, *Halimeda tuna*, *Arrainvilla rawsonii*, *Codium iyengarii*, and *Ulva fasciata* green algae, respectively [58]. Dopamine is an allelopathic compound produced by the green algae *Ulvaria obscura* that negatively affects the development of coexisting aquatic animals [46,59]. The *U. obscura* is a common Chlorophyta that often forms the green tides in the northeastern Pacific [47], where it can coexist with other green macroalgal species such as *Ulva lactuca*, *U. prolifera*, and *U. linza*. Nelson et al. [47] hypothesized that dopamine is responsible for some harmful effects observed in coexisting aquatic animals. Paul and Fenical [60] showed that halimedatrial can completely inhibit the motility of sea urchin (*Lytechinus pictus*) sperm. Halimedatrial is a diterepene trialdhyde isolated from various species of the genus *Halimeda* (Chlorophyta) such as *H. tuna*, *H. opuntia*, *H. incrassata*, *H. simulans*, *H. scabra*, and *H. copiosa*. This compound is also toxic toward reef damselfishes (*Eupomacentrus planifrons* and *Dascyllus aruanus*) and significantly reduces feeding in these herbivorous fishes [60].

Marine red algae are the most important source of many biologically active compounds (Table 4). For instance, the Rhodophyta *Callophycus serratus*, *Plocumium carttilagineum*, *Portieria hornemanii*, *Laurencia okamurai*, and *Laurencia viridis* are sources of bromophycolides C-I, furoplocamioid C, halmon, laurinterol, and thyresenol A-B compounds, respectively [58]. Moreover, tichocarpols A and B are compounds isolated from the red alga *Tichocarpus crinitus*, and they exhibit antifeedant activity against the sea urchin *Strongylocentrotus intermedius* [36]. Williamson et al. [33] described that the floridoside-isethionic acid complex produced and released by *Delisea pulchra* induced metamorphosis in the *Holopneustes purpurascens* sea urchin.

Many bioactive metabolites with different biological activities have also been isolated from Ochrophyta (Table 4). Brown algae species such as *Bifurcaria bifurcata*, *Dictyota dichotoma*, *Cystoseira tamariscifolia*, *Lobophora variegate*, *Sargassum siliquastrum*, and *Turbinaria ornate* can produce compounds such as bifurcadiol, dictyotins, meroditerpenoid, lobophorolide, sargachromanols, and turbinaric acid, respectively [58]. Tanaka and Higa [32] noted that *Dictyota spinulosa* are not commonly eaten by the herbivorous fish *Tilapia mossambica* because it produces allelopathic diterpene. Similarly, two diterpenoids (dictyterepenoids A and B), which were isolated from the *Dilophus okamurae* brown algae, display antifeedant activity against the *Haliotis discus hannai* abalone [34]. Furthermore, *Dictyota pfaffi* brown algae also produce antifeedant compounds (diterpenoid 10,18-diacetoxy-8-hydroxy-2,6-dolabelladiene) against herbivores (sea urchins and fishes) [35]. Gerwick et al. [30] showed that stypoldione isolated from *Stypopodium zonale* brown algae exhibits ichthyotoxic activity on herbivorous reef-dwelling fish *Eupomcentrus leucostictus*. Two years later, Gerwick and Fenical [31] described other compounds obtained from this brown alga, including stypotriol, stypodiol, epistypodiol, epitaondiol, 2-(geranyl-geranyl)-5-methyl-1,4-benzohydroquinone, 2-(geranyl-geranyl)-5-methyl-l,4-benzoquinone, taondiol, and atomaric acid, which showed toxic effects toward *E. leucostictus*. These authors also reported that stypoldione from *S. zonale* is a potent inhibitor of cell division in the fertilized eggs of the sea urchin *Strongylocentrotus purpuratus*.

Although freshwater and brackish macroscopic green algae (Chlorophyta and Charophyta) can produce allelochemicals with interesting properties [61,62,63,64], they have not been widely investigated (Table 4). Wium-Andersen et al. [61,62] showed that freshwater *Chara globularis* (Charophyta, Charophyceae) negatively affects natural phytoplankton assemblages via two sulfuric compounds: dithiolane and trithiane. Anthoni et al. [63] isolated charamin, which has strong antibiotic activity, from *C. globularis*. More recently, Korzeniowska et al. [64] identified nine phenolic compounds obtained from freshwater *Cladophora glomerata* (Chlorophyta, Ulvophyceae) however, the activity of these compounds on aquatic animals has not been tested (Table 4).

**Table 4:** Macroalgae capable of producing bioactive compounds against other heterotrophic organisms (not only competing and herbivorous), location of their environmental occurrence, name of compounds, and their effect on target organisms.

Phylum/Species	Habitat	Compound	Activity	References
**Green Algae (Chlorophyta)**				
*Avrainvillea nigricans*	marine	Nigricanosides A–B	Antimitotic agent	Williams et al. [65]
*Avrainvillea nigricans*	marine	Hydroxyisoavrainvilleol	Protein tyrosine phosphate 1B inhibitors (PTP1B)	Colon et al. [66]
*Avrainvillea rawsonii*	marine	Isorawsonol	Cytotoxic and immunosuppressive activities	Chen et al. [67]
*Bryopsis* sp.	marine	Kahalalide F	Cytotoxic and immunosuppressive activities	Hamann and Scheuer [68]
*Bryopsis* sp.	marine	Kahalalide P	Cytotoxic and immunosuppressive activities	Dmitrenok et al. [69]
*Caulerpa racemosa*	marine	Sulfoquinovosyldiacylglycerol	Antiviral activity	Wang et al. [70]
*Caulerpa taxifolia*	marine	Caulerpals A–B	Anti-fungal activity	Aguilar-Santos [71]
*Chara globularis*	freshwater	Charamin	Antibiotic activity	Anthoni et al. [63]
*Chara globularis*	freshwater	Dithiolane, Trithiane	Antialgal activity	Wium-Andersen et al. [61]
*Cladophora glomerata*	freshwater	Gallic acid, Chlorogenic acid, Syringic acid, *p*-coumaric acid, Myricetin, 3,4-dihydroxybenzoic acid, Vanillic acid, 4-hydroxybenzoic acid, Rutin	Unknown	Korzeniowska et al. [64]
*Codium iyengarii*	marine	Lyengaroside	Antibacterial activity	Ali et al. [72]
*Cymopolia barbata*	marine	Cymobarbatol, 4-isocymobarbatol	Antimutagenic activity	Wall et al. [73]
*Halimedatuna*, *Halimedaopuntia*, *Halimedaincrassata*, *Halimedasimulans*, *Halimedascabra*, *Halimedacopiosa*	marine	Halimedatrial	Cytotoxic and antimicrobial activities	Paul and Fenical [60]
*Halimeda tuna*	marine	Halitunal	Antibacterial activity	Koehn et al. [74]
*Halimeda* sp.	marine	Halimedatrial	Antimicrobial and cytotoxic properties	Paul and Fenical [75]
*Penicillus capitatus*	marine	Capisterones A–B	Anti-fungal activity	Puglisi et al. [76]
*Tydemania expeditionis*	marine	Cycloartenol disulfates	Cytotoxic and immunosuppressive activities	Govindan et al. [77]
*Ulva* (*Enteromorpha*) *intestinals*	marine	Penostatins A–H	Cytotoxic and immunosuppressive activities	Takahashi et al. [78], Iwamoto et al. [79,80]
*Ulva* (*Enteromorpha*) *intestinalis*	marine	Cytochalasans, penochalasins A–H	Cytotoxic activity	Numata et al. [81]
*Ulva* (*Enteromorpha*) *intestinalis*	marine	Chaetoglobosin	Cytotoxic activity	Iwamoto et al. [82]
*Ulva* (*Enteromorpha*) *intestinals*	marine	Communesins A–B	Cytotoxic and immunosuppressive activities	Numata et al. [83]
*Ulva lactuca*	marine	3-0-*β*-d-glucopyranosy-lstigmasta-5,25-diene	Anti-inflammatory substances	Awad et al. [84]
*Ulvaria obscura*	marine	Dopamine	Feeding-deterrent substances	Tocher and Craigie [59], Van Alstyne et al. [46]
**Red Algae (Rhodophyta)**				
*Beckerella* (*Gelidium*) *subcostatum*	marine	Bromo- beckerelide, epimer, chlorobeckerelide	Antimicrobial activity	Ohta [85]
*Callophycus serratus*	marine	Bromophycolides A–B	Cytotoxic activity	Kubanek et al. [86]
*Callophycus serratus*	marine	Bromophycolides C–I	Cytotoxic activity	Kubanek et al. [87]
*Callophycus serratus*	marine	Callophycoic acids A–H, diterpene-phenols, callophycols A–B	Antibacterial, antimalarial, anti-tumor and antifungal activity	Lane et al. [88]
*Chondria armata*	marine	Isodomic acid A–C	Insecticidal activity	Maeda et al. [89]
*Chondria atropurpurea*	marine	Chondriamide C, 3-indolacrylamide	Anthelmintic activity	Davyt et al. [90]
*Chondria oppositiclada*	marine	Cycloeudesmol	Antibacterial activity	Fenical and Sims [91]
*Delisea pulchra*	marine	Floridoside-isethionic acid complex	Induction of animal metamorphosis	Williamson et al. [33]
*Digenea simplex*	marine	α-alko-kainic acid	Neurophysiological activity	Biscoe et al. [92], Ferkany and Coyle [93]
*Gracilaria asiatica*	marine	Cerebroside gracilarioside, ceramides gracilamides A–B	Cytotoxic activity	Sun et al. [94]
*Gigartina tenella*	marine	Sulquinovosyldiacylglycerol: KM043	Antiviral activity	Ohata et al. [95]
*Jania rubens*	marine	Deoxyparguerol-7-acetate	Anthelmintic activity	Awad [96]
*Laurencia brongniartii*	marine	Polybromoindoles	Antimicrobial activity, cytotoxic activity	Carter et al. [97], El Gamal et al. [98]
*Laurencia brongniartii*	marine	Brominated indoles	Antibacterial activities	Carter et al. [97]
*Laurencia elata*	marine	Elatol	Antibacterial activities	Sims [99]
*Laurencia obtusa*	marine	Teurilene, thyrsiferyl 23-acetate	Cytotoxic activity	Suzuki et al. [100]
*Laurencia obtusa*	marine	3,7-dihydroxydihydrolaurene, perforenol B	Cytotoxic activity	Kladi et al. [101]
*Laurencia obtusa*	marine	Neorogioldiol B, prevezol B–D	Cytotoxic activity	IIopoulou et al. [102]
*Laurencia obtusa*	marine	Iso-obtusol	Antibacterial activities	Gonzalez et al. [103,104]
*Laurencia obtusa*	marine	Sesquiterpene	Antimalarial activity	Topeu et al. [105]
*Laurencia pinnatifida*	marine	Dehydrothyrsiferol, thyresenol A and B	Cytotoxic activity	Norte et al. [106], Pec et al. [107]
*Laurancia pinnata*	marine	Laurepinacine, isolaurepinnacin	Insecticidal activity	Fukuzawa and Masamune [108]
*Laurencia mariannensis*	marine	Brominated diterpene, 10-hydroxykahukuene B, 9-deoxyelatol, isoda-ctyloxene A, C15-acetogenin, laurenmariallene, sesquiterpenes	Antibacterial activities	Gonzalez et al. [109]
*Laurencia nidifica*	marine	Laurinterol, isolaurinterol, aplysin, α-bromocuparene	Insecticidal and repellent activities	Ishii et al. [110]
*Laurencia nipponica*	marine	(Z)-Laureatin, (Z)-isolaureatin, deoxyprepacifenol	Insecticidal activity	Watanabe et al. [111], El Sayed et al. [112]
*Laurencia okamurae*	marine	Laurinterol	Cytotoxic activity	Moon-Moo et al. [113]
*Laurencia scoparia*	marine	*β*-bisabolene sesquiterpenes	Anthelmintic activity	Davyt et al. [114]
*Laurencia tristicha*	marine	Cholest-5-en-3β,7α-diol Debromoepiaplysinol	Cytotoxic activity	Sun et al. [115]
*Laurencia venusta*	marine	Venustatriol	Antiviral activity	Sakemi et al. [116]
*Laurencia yonaguniensis*	marine	Neoirietetraol	Cytotoxic activity	Takahashi et al. [117]
*Lophocladia* sp.	marine	Lophocladine B	Cytotoxic activity	Gross et al. [118]
*Murrayella periclados*	marine	12S-hydroxyeicosapentaenoic acid	Lipooxygenase inhibitor	Bernari and Gerwick [119]
*Odonthalia corymbifera*	marine	Bromophenols	Inhibition of isocitrate lyase enzyme	Lee et al. [120]
*Peyssonnelia* sp.	marine	Avarol	Antiviral activity	Talpir et al. [121]
*Plocamium corallorhiza*	marine	Plocaralides B–C	Cytotoxic activity	Knott et al. [122]
*Plocamium telfairiae*	marine	Telfairine	Insecticidal activity	Watanabe et al. [123]
*Ptilota filicina*	marine	Ptiollodene	Lipo-oxygenase inhibitor	Lopez and Gerwick [124]
*Symphyocladia latiuscula*	marine	Tasipeptins A–B	Aldose reductase inhibitors activity	Wang et al. [125]
*Vidalia obtusiloba*	marine	Vidalols A–B	Anti-inflammatory activity	Wiemer et al. [126]
**Brown Algae (Ochrophyta)**				
*Chondria oppositiclada*	marine	Cycloeudesmol	Antibacterial activity	Fenical and Sims [91]
*Cystoseira crinita*	marine	Meroterpenoids	Free radical scavenger and antioxidant activities	Fisch et al. [127]
*Cystoseira myrica*	marine	Hydroazulene diterpenes	Cytotoxic activity	Ayyad et al. [128]
*Cystoseira tamariscifolia*	marine	Methoxybifurcarenone	Antifungal and antibacterial activity	Bennamara et al. [129]
*Cystophora siliquosa*	marine	Cystophorene	Sperm-attractants pheromone	Muller et al. [130]
*Dictyopteris undulata*	marine	Yahazunol	Antimicrobial activity	Ochi et al. [131]
*Dictyopteris undulata*	marine	Cyclozonarone	Feeding-deterrent activity	Kurata et al. [132]
*Dictyopteris zonarioides*	marine	Zonarol, isozonarol	Antifungal activity	Fenical et al. [133]
*Dictyota pfaffi*	marine	10,18-diacetoxy—8-hydroxy 2,6-dollabeladiene (dolabellane 1)	Antiviral activity	Barbosa et al. [35,134]
*Dictyota spinulosa*	marine	Hydroxydictyodial	Feeding-deterrent substances	Tanaka and Higa [32]
*Dictyota* sp.	marine	Dolabellane diterpenes	Cytotoxic activity	Tringali et al. [135]
*Dilophus okamurae*	marine	Dictyterepenoids A–B	Antifeedent activity	Suzuki et al. [34]
*Ecklonia cava*	marine	Fucodiphlorethol G	Antioxidant activity	Ham et al. [136]
*Ecklonia stolonifera*	marine	Phloroglucinol, eckstolonol, eckol, phlorofucofuroeckol A, dieckol	Hepatoprotective activity	Kang et al. [137]
*Giffordia mitchelliae*	marine	Giffordene	Gamete-attracting pheromone	Boland et al. [138]
*Hizikia fusiformis*	marine	Arsenic-containing ribofuranosides	Cytotoxic activity	Edmonds et al. [139]
*Hormosira banksii*	marine	Hormosirene	Sperm-attractants pheromone	Muller et al. [130]
*Leptosphaeria* sp.	marine	Leptosins M, MI, N, N1	Cytotoxic activity	Yamada et al. [140]
*Lobophora variegata*	marine	Lobophorolide	Antifungal activity	Kubanek et al. [141]
*Notheia anomala*	marine	*cis* dihydroxyte-trahydrofuran	Nematocidal activity	Capon et al. [142]
*Osmundaria serrata*	marine	Lanosol enol ether	Antifungal and antibacterial activity	Barreto and Meyer [143]
*Perithalia caudata*	marine	Caudoxirene	Gamete-releasing, gamete-attracting pheromone	Muller et al. [144]
*Pelvetia siliquosa*	marine	Fucosterol	Anti-diabetic activity	Lee et al. [145]
*Sargassum siliquastrum*	marine	Sargachromanols A–P	Antioxidant activity	Jang et al. [146]
*Sargassum tortile*	marine	Dihydroxysargaquinone	Cytotoxic activity	Numata et al. [147]
*Sargassum tortile*	marine	Hydroxysargaquinone, sargasal-I-II	Cytotoxic activity	Numata et al. [148]
*Sargassum thunbergii*	marine	Thunbergols A–B	Scavenging activities, antioxidant activity	Seo et al. [149]
*Sargassum thunbergii*	marine	Sargothunbergol A	Antioxidant activity	Seo et al. [150]
*Sargassum thunbergii*	marine	Diacylglycerols	Antifungal activity	Kim et al. [151]
*Stypopodium flabelliforme*	marine	Isoepitaondiol	Insecticidal activity	Rovirosa et al. [152]
*Stypopodium zonale*	marine	Stypolactone	Cytotoxic activity	Dorta et al. [153]
*Stypopodium zonale*	marine	Stypotriol, stypoldione	Ichthyotoxic activity	Gerwick et al. [30]
*Stypopodium zonale*	marine	Stypoquinonic acid, taondiol, atomaric acid	Antimicrobial activity	Wessels et al. [154]
*Stypopodium zonale*	marine	Stypoldione, stypotriol, stypodiol, epistypodiol, epitaondiol	Ichthyotoxic activity, cytotoxic activity	Gerwick and Fenical [31]
*Taonia atomaria*	marine	Taondiol	Antimicrobial activity, cytotoxic activity	Othmani et al. [155]
*Taonia atomaria*	marine	Tetraprenyl benzoquinone sargaquinone	Anti-inflammatory activity	Tziveleka et al. [156]
*Taonia atomaria*	marine	Meroditerpenes atomarianones A–B	Cytotoxic activity	Abatis et al. [157]
*Turbinaria ornata*	marine	Turbinaric acid	Cytotoxic activity	Asari et al. [158]

Allelopathic activity is likely to involve more than one mechanism. Allelochemicals may indirectly influence multiple physiological processes, and phenotypic reactions to a particular compound may result from secondary effects [159]. Different mechanisms function depending on whether allelopathy occurs in open water (pelagic zone) or is associated with substrate (benthic habitats) [12], and many biotic and abiotic factors influence the severity of allelopathic interactions. Macroalgae secrete allelochemicals by direct contact or through masses of water; this is especially facilitated due to the small molecules that make up these compounds. In the case of direct contact, this happens through compounds contained in epidermal glands, secretory trichomes, or in other ways associated with the plant surface [20]. Allelopathic compounds can alter the permeability and fluidity of cell membranes and disturb the activity of membrane proteins and intracellular enzymes, particularly those that build antioxidant systems [160]. Moreover, allelochemicals can also cause oxidative damage and activation of antioxidant mechanisms [161]. In addition, allelopathic compounds have been observed to affect photosynthesis [162] and have been influenced by environmental factors (temperature, light intensity, water availability, CO_2_ concentration, and microorganisms) [163]. A potential site of action for allelochemicals is the mitochondria because mitochondrial respiration is essential for the production of ATP, which is used in metabolic processes, for example, macromolecular synthesis [164].

Macroalgae are a rich source of highly bioactive secondary metabolites that may have potential applications. Macroalgae biomass are widely used in the chemical, food, agriculture, cosmetics, pharmacy, and medicine industries. Macroalgae are also rich in various biologically active substances valued for their, among others, antimicrobial, anti-inflammatory, antioxidant, antifungal, cytotoxic, and insecticidal activity [58,165]. Additionally, allelochemicals from macroalgae on herbivores may have potential in limiting the negative expansion of invasive species worldwide (Table 4). This research highlights the possibility of exploiting the allelopathic potential of macroalgae in commercial aquaculture. The characterization of macroalgal allelochemicals as well as their mode of action are still poorly understood. In addition, most studies have focused on the activity of allelopathic compounds derived from marine macroalgae. Therefore, future research should also include the isolation and identification of allelopathic compounds from freshwater and brackish macroalgae.

## 6. The Limitation of Macroalgae-Herbivores Interactions

Herbivores have a great influence on macroalgae in all water types [166]. A multidisciplinary ecophysiological approach is required to study macroalgae-herbivores interactions in combination with other mechanisms affecting plants. Most macroalgae show some form of anti-herbivore strategy. These relate to physical features that allow escape or chemical features that allow for defense, e.g., by release of secondary metabolites [167]. Thus, research can include both the ecological and molecular levels. The production of allelochemicals has been shown to increase under certain conditions. Del Monaco et al. [25] suggested that increasing ocean acidification can cause advantages to seaweeds over corals and that ocean acidification may enhance the allelopathy of certain macroalgae. Conversely, Ritson-Williams et al. [24] described that increased seawater temperatures made larvae more susceptible to a concurrent local stressor disrupting a key process of coral reef recovery and resilience. The process of synthesizing molecules of allelopathic compounds is controlled by a number of physiological, chemical, and spatial-temporal variables [8]. The toxicity gradient may be related to habitat complexity. More toxic macroalgae extracts are found in reef sites and in rocky intertidal environments. The presence or absence of toxicity was also observed depending on sample collection site and climate [8]. Additionally, allelopathy can only be effective when plants are under stress caused by other mechanisms, such as deprivation of water or intense competition for both nutrients or light. The target plant is also more susceptible to phytotoxins when under stress [168]. Furthermore, bacteria associated with the target or donor organism may metabolize the excreted allelochemicals [12]. It is important to pay attention and avoid misunderstandings, especially in distinguishing allelopathy from any other competitive or noncompetitive relationship [12]. A small number of authors model allelopathic interactions using field or experimental data e.g., [169,170,171,172,173,174]. Such studies usually must oversimplify processes, which may not always be satisfactory. Thus, the method for testing the effects of allelopathic macroalgae on target organisms should be chosen carefully. Macroalgae extracts and exudates provide an environment that is distant from the environmental conditions of the test organisms while experiments in mesocosms or arranged co-culturing experiments are closer to the conditions of natural occurrence of macroalgae and studied animals and are thus more reflective of naturally occurring processes.

## 7. Conclusions

Macroalgae are the sources of many harmful allelopathic compounds, which are synthesized as a defense strategy against competitors and predators. Macroalgae can produce inhibitory compounds affecting competitors for the Cnidaria phylum on tropical reefs. The strongest negative effect against Cnidaria occur from macroalgae of the genus *Bryopsis*, *Chlorodesmis*, *Halimeda,* and *Rhiphilia* (Chlorophyta, green algae); *Amansia*, *Asparagopsis*, *Callophycus*, *Endosiphonia*, *Galaxaura*, *Phacelocarpus*, and *Plocamium* (Rhodophyta, red algae); as well as *Sphacelaria* (Ochrophyta, brown algae). Several studies have also demonstrated the negative effects of macroalgae on predators (Mollusca, Annelida, Echinodermata, Arthropoda, and Chordata species) upon ingestion. *Chaetomorpha*, *Codium*, and *Ulva* (green algae); *Grateloupia* and *Polysiphonia* (red algae); and *Desmarestia* and *Laminaria* (brown algae) strongly inhibit Annelida development. Furthermore, red (*Tichocarpus* sp.) and brown (*Dictyota* sp. and *Stypopodium* sp.) algae negatively affect species belonging to Echinodermata. Some studies also examined negative effects of *Ulvaria obscura* (green algae) on Arthropoda species. The strong negative influence of the red algae *Chondriopsis* sp. on Chordata, and brown algae *Dilophus* sp. on Mollusca has been demonstrated. Although the term macroalgal allelopathy refers to the effects of substances produced by macroalgae that can be both harmful and beneficial to target organisms, positive effects of algae on aquatic animals are extremely rare. Only certain species of green (*Chlorodesmis* sp., *Ulva* sp., and *Ulvaria* sp.), red (*Delisea* sp.), and brown algae (*Lobophora* sp.) positively affect certain Cnidaria, Mollusca, and Echinodermata species. In addition, the allelopathic activity of macroalgae can change according to the taxonomic position of the donor and target organisms, as well as their habitat. However, most studies have focused on the allelopathic effects of macroalgae in marine environments. Therefore, future studies should consider the nature of released substances and their effect on target organisms of freshwater and brackish macroalgae. Furthermore, the allelopathy phenomenon of macroalgae in aquatic ecosystems should be further studied considering both scientific and commercial aspects.

## Figures and Tables

**Table 1:** Examples of allelopathic activity of green algae against competitors and herbivores.

**Donor Chlorophyta**	**Target Organism—Cnidaria**	**Effect**	**References**
*Bryopsis corymbose*	*Pocillopora damicornis*	−	Lee et al. [28]
*Bryopsis* sp.	*Pocillopora acuta*	−	Fong et al. [39]
*Chlorodesmis fastigiata*	*Acropora millepora*	+/0	Birrell et al. [40]
*Chlorodesmis fastigiata*	*Acropora aspera*	−	Bonaldo and Hay [23]
	*Pocillopora damicornis*	−	
	*Porites cylindrica*	−	
	*Porites lobata*	−	
*Chlorodesmis fastigiata*	*Acropora intermedia*	−	Del Monaco et al. [25]
*Chlorodesmis fastigiata*	*Phialophora verrucosa*	−	Longo and Hay [26]
*Chlorodesmis fastigiata*	*Acropora millepora*	−	Rasher et al. [22]
	*Montipora digitata*	−	
	*Pocillopora damicornis*	−	
*Chlorodesmis fastigiata*	*Acropora millepora*	−	Ritson-Williams et al. [24]
	*Montipora digitata*	−	
	*Pocillopora damicornis*	−	
*Chlorodesmis fastigiata*	*Acropora cuneata*	−	Tanner [21]
	*Acropora hrueggemanni*	−	
	*Acropora pnlifera*	−	
	*Pocillopora damicornis*	−	
*Halimeda opuntia*	*Pocillopora damicornis*	−	Lee et al. [28]
*Halimeda tuna*	*Montastraea faveolate*	−	Morrow et al. [52]
	*Porites astreoides*	0	
*Halimeda* sp.	*Acropora cuneata*	−	Tanner [21]
	*Acropora hrueggemanni*	−	
	*Acropora pnlifera*	−	
	*Pocillopora damicornis*	−	
*Rhiphilia pencilloides*	*Porites rus*	−	Andras et al. [51]
**Donor Chlorophyta**	**Target Organism—Mollusca**	**Effect**	**References**
*Ulva australis*	*Haliotis rubra*	+	Huggett et al. [49]
*Ulva compressa*	*Crassostrea virginica*	−	Green-Gavrielidis et al. [38]
*Ulva compressa*	*Haliotis rubra*	+	Huggett et al. [49]
*Ulva fenestrata*	*Crassostrea gigas*	−	Nelson et al. [47]
*Ulva lactuca*	*Crassostrea virginica*	−	Green-Gavrielidis et al. [38]
*Ulvaria lactuca*	*Crassostrea gigas*	−	Nelson and Gregg [48]
*Ulva lens*	*Crassostrea gigas*	−	Nelson et al. [47]
*Ulvaria obscura*	*Haliotis rubra*	+	Huggett et al. [49]
*Ulva obscura*	*Crassostrea virginica*	−	Green-Gavrielidis et al. [38]
*Ulvaria obscura*	*Crassostrea gigas*	−	Nelson and Gregg [48]
*Ulva* sp.	*Haliotis rufescens*	+	Muñoz et al. [50]
**Donor Chlorophyta**	**Target Organism—Annelida**	**Effect**	**References**
*Chaetomorpha* sp.	*Sabellaria cementarium*	−	Warkus et al. [41]
	*Spinoidae* sp.	−	
*Codium fragile*	*Sabellaria cementarium*	−	Warkus et al. [41]
	*Spinoidae* sp.	−	
*Ulva* (*Enteromorpha*) sp.	*Sabellaria cementarium*	−	Warkus et al. [41]
	*Spinoidae* sp.	−	
*Ulva lactuca*	*Sabellaria cementarium*	−	Warkus et al. [41]
	*Spinoidae* sp.	−	
**Donor Chlorophyta**	**Target Organism—Arthropoda**	**Effect**	**References**
*Ulvaria obscura*	*Cancer oregonensis*	0/−	Van Alstyne et al. [46]
	*Metacarcinus magister*	0/−	
**Donor Chlorophyta**	**Target Organism—Chordata**	**Effect**	**References**
*Anadyomene stellata*	*Carassius auratus auratus*	0/−	Alvarez-Hernández et al. [8]
*Caulerpa cupressoides*	*Carassius auratus auratus*	0/−	Alvarez-Hernández et al. [8]
*Caulerpa paspaloides*	*Carassius auratus auratus*	0/−	Alvarez-Hernández et al. [8]
*Caulerpa racemosa*	*Carassius auratus auratus*	0/−	Alvarez-Hernández et al. [8]
*Chaetomorpha antennina*	*Carassius auratus auratus*	0/−	Alvarez-Hernández et al. [8]
*Penicillus capitatus*	*Carassius auratus auratus*	0/−	Alvarez-Hernández et al. [8]

Note: − means inhibiting effects, + means stimulating effect, 0—means lack of effect.

**Table 2:** Examples of allelopathic activity of red algae against competitors and herbivores.

**Donor Rhodophyta**	**Target Organism—Cnidaria**	**Effect**	**References**
*Amphiroa crassa*	*Acropora millepora*	−	Rasher et al. [22], Ritson-Williams et al. [24]
	*Montipora digitata*	0	
	*Pocillopora damicornis*	−	
*Amansia glomerata*	*Acropora intermedia*	−	Del Monaco et al. [25]
*Amansia rhodantha*	*Phialophora verrucosa*	−	Longo and Hay [26]
*Asparagopsis taxiformis*	*Phialophora verrucosa*	−	Longo and Hay [26]
*Callophycus densus*	*Porites rus*	−	Andras et al. [51]
*Chondrophycus poiteaui*	*Porites astreoides*	0	Kuffner et al. [42]
*Endosiphonia horrida*	*Pocillopora acuta*	−	Fong et al. [39]
*Galaxaura filamentosa*	*Acropora millepora*	−	Rasher et al. [22], Ritson-Williams et al. [24]
	*Montipora digitata*	−	
	*Pocillopora damicornis*	−	
*Galaxaura filamentosa*	*Acropora aspera*	−	Bonaldo and Hay [23]
	*Pocillopora damicornis*	−	
	*Porites cylindrica*	−	
	*Porites lobata*	−	
*Hypnea pannosa*	*Pocillopora acuta*	0	Fong et al. [39]
*Liagora* sp.	*Acropora millepora*	−	Rasher et al. [22], Ritson-Williams et al. [24]
	*Montipora digitata*	0	
	*Pocillopora damicornis*	−	
*Phacelocarpus neurymenioides*	*Porites rus*	−	Andras et al. [51]
*Plocamium pacificum*	*Porites rus*	−	Andras et al. [51]
*Peyssonnelia* sp.	*Acropora cuneata*	−	Tanner [21]
	*Acropora hrueggemanni*	−	
	*Acropora pnlifera*	−	
	*Pocillopora damicornis*	0/−	
**Donor Rhodophyta**	**Target Organism—Annelida**	**Effect**	**References**
*Grateloupia turu turu*	*Sabellaria cementarium*	−	Warkus et al. [41]
	*Spinoidae* sp.	−	
*Polysiphonia denudata*	*Sabellaria cementarium*	−	Warkus et al. [41]
	*Spinoidae* sp.	−	
**Donor Rhodophyta**	**Target Organism—Echinodermata**	**Effect**	**References**
*Delisea pulchra*	*Holopneustes purpurascens*	+	Williamson et al. [33]
*Tichocarpus crinitus*	*Strongylocentrotus intermedius*	−	Ishii et al. [36]
**Donor Rhodophyta**	**Target Organism—Chordata**	**Effect**	**References**
*Acanthophora spicifera*	*Carassius auratus auratus*	0/−	Alvarez-Hernández et al. [8]
*Amphiroa beauvoisii*	*Carassius auratus auratus*	0/−	Alvarez-Hernández et al. [8]
*Centroceras clavulatum*	*Carassius auratus auratus*	0/−	Alvarez-Hernández et al. [8]
*Ceramium nitens*	*Carassius auratus auratus*	0/−	Alvarez-Hernández et al. [8]
*Chondria littoralis*	*Carassius auratus auratus*	0	Alvarez-Hernández et al. [8]
*Chondriopsis dasyphylla* f. *pyrifera*	*Carassius auratus auratus*	−	Alvarez-Hernández et al. [8]
*Crassiphycus caudatus* (*Gracilaria caudata*)	*Carassius auratus auratus*	0	Alvarez-Hernández et al. [8]
*Dermonema virens*	*Carassius auratus auratus*	0	Alvarez-Hernández et al. [8]
*Digenea simplex*	*Carassius auratus auratus*	0	Alvarez-Hernández et al. [8]
*Gracilaria cervicornis*	*Carassius auratus auratus*	0/−	Alvarez-Hernández et al. [8]
*Gracilaria tikvahiae*	*Carassius auratus auratus*	0/−	Alvarez-Hernández et al. [8]
*Grateloupia filicina*	*Carassius auratus auratus*	0	Alvarez-Hernández et al. [8]
*Hypnea musciformis*	*Carassius auratus auratus*	0/−	Alvarez-Hernández et al. [8]
*Hypnea spinella*	*Carassius auratus auratus*	0/−	Alvarez-Hernández et al. [8]
*Laurencia obtusa*	*Carassius auratus auratus*	0/−	Alvarez-Hernández et al. [8]
*Liagora ceranoides*	*Carassius auratus auratus*	0/−	Alvarez-Hernández et al. [8]
*Tayloriella dictyurus*	*Carassius auratus auratus*	0	Alvarez-Hernández et al. [8]
*Yuzurua poiteaui* var. *gemmifera*	*Carassius auratus auratus*	0	Alvarez-Hernández et al. [8]

Note: − means inhibiting effects, + means stimulating effect, 0—means lack of effect.

**Table 3:** Examples of allelopathic activity of brown algae against competitors and herbivores.

**Donor Ochrophyta**	**Target Organism—Cnidaria**	**Effect**	**References**
*Dictyota bartayresiana*	*Phialophora verrucosa*	−	Longo and Hay [26]
*Dictyota bartayresiana*	*Acropora millepora*	−	Rasher et al. [22], Ritson-Williams et al. [24]
	*Montipora digitata*	−	
	*Pocillopora damicornis*	−	
*Dictyota bartayresiana*	*Acropora cervicornis*	0	Ritson-Williams et al. [27]
	*Acropora palmata*	−	
	*Pseudodiploria strigosa*	0	
*Dictyota cervicornis*	*Acropora intermedia*	−	Del Monaco et al. [25]
*Dictyota menstrualis*	*Porites astreoides*	−	Olsen et al. [56]
*Dictyota pinnatifida*	*Porites astreoides*	−	Paul et al. [55]
*Dictyota pulchella*	*Porites astreoides*	−	Paul et al. [55]
*Dictyota pulchella*	*Acropora cervicornis*	0	Ritson-Williams et al. [27]
	*Acropora palmata*	−	
	*Pseudodiploria strigosa*	0	
*Dictyota* sp.	*Montastraea faveolate*	0/−	Morrow et al. [52]
	*Porites astreoides*	0/−	
*Dictyota* sp.	*Briareum asbestinum*	−	Kuffner et al. [42]
	*Porites astreoides*	−	
*Lobophora abscondita*	*Acropora muricate*	−	Vieira et al. [57]
	*Montipora hirsute*	0	
	*Porites cylindrica*	0	
	*Stylophora pistillata*	−	
*Lobophora crassa*	*Acropora muricate*	−	Vieira et al. [57]
	*Montipora hirsute*	0	
	*Porites cylindrica*	0	
	*Stylophora pistillata*	−	
*Lobophora dimorpha*	*Acropora muricate*	−	Vieira et al. [57]
	*Montipora hirsute*	0	
	*Porites cylindrica*	0	
	*Stylophora pistillata*	−	
*Lobophora hederacea*	*Acropora muricate*	−	Vieira et al. [57]
	*Montipora hirsute*	0	
	*Porites cylindrica*	0	
	*Stylophora pistillata*	−	
*Lobophora monticola*	*Acropora muricate*	−	Vieira et al. [57]
	*Montipora hirsute*	0	
	*Porites cylindrica*	0	
	*Stylophora pistillata*	−	
*Lobophora nigrescens*	*Acropora muricate*	−	Vieira et al. [57]
	*Montipora hirsute*	0	
	*Porites cylindrica*	0	
	*Stylophora pistillata*	−	
*Lobophora rosacea*	*Acropora muricate*	−	Vieira et al. [57]
	*Montipora hirsute*	0	
	*Porites cylindrica*	0	
	*Stylophora pistillata*	−	
*Lobophora undulata*	*Acropora muricate*	−	Vieira et al. [57]
	*Montipora hirsute*	0	
	*Porites cylindrica*	0	
	*Stylophora pistillata*	−	
*Lobophora variegata*	*Acropora millepora*	+	Birrell et al. [40]
*Lobophora variegata*	*Briareum asbestinum*	−	Kuffner et al. [42]
	*Porites astreoides*	−	
*Lobophora variegata*	*Montastraea faveolate*	−	Morrow et al. [52]
	*Porites astreoides*	−	
*Lobophora* sp.	*Pocillopora acuta*	−	Fong et al. [39]
*Lobophora* sp.	*Acropora cervicornis*	−	Ritson-Williams et al. [27]
	*Acropora palmata*	−	
	*Pseudodiploria strigosa*	0	
*Padina boryana*	*Acropora millepora*	−	Rasher et al. [22], Ritson-Williams et al. [24]
	*Montipora digitata*	0	
	*Pocillopora damicornis*	−	
*Padina minor*	*Pocillopora damicornis*	0	Lee et al. [28]
*Padina* sp.	*Acropora millepora*	−	Birrell et al. [40]
*Sargassum polycystum*	*Acropora millepora*	−	Ritson-Williams et al. [24]
	*Montipora digitata*	0	
	*Pocillopora damicornis*	−	
*Sargassum* sp.	*Pocillopora damicornis*	−	Lee et al. [28]
*Sphacelaria* sp.	*Acropora millepora*	−	Webster et al. [29]
*Turbinaria conoides*	*Acropora millepora*	0	Rasher et al. [22]
	*Montipora digitata*	0	
	*Pocillopora damicornis*	0	
*Turbinaria conoides*	*Acropora millepora*	−	Ritson-Williams et al. [24]
	*Montipora digitata*	0	
	*Pocillopora damicornis*	−	
*Turbinaria ornata*	*Phialophora verrucosa*	0	Longo and Hay [26]
*Turbinaria ornata*	*Acropora cuneata*	−	Tanner [21]
	*Acropora hrueggemanni*	−	
	*Acropora pnlifera*	−	
	*Pocillopora damicornis*	−	
**Donor Ochrophyta**	**Target Organism—Mollusca**	**Effect**	**References**
*Dilophus okamurae*	*Haliotis discus hannai*	−	Suzuki et al. [34]
**Donor Ochrophyta**	**Target Organism—Annelida**	**Effect**	**References**
*Desmarestia viridis*	*Sabellaria cementarium*	−	Warkus et al. [41]
	*Spinoidae* sp.	−	
*Laminaria* sp.	*Sabellaria cementarium*	−	Warkus et al. [41]
	*Spinoidae* sp.	−	
**Donor Ochrophyta**	**Target Organism—Echinodermata**	**Effect**	**References**
*Dictyota pfaffi*	*Lytechinus variegates*	−	Barbosa et al. [35]
*Ecklonia radiata*	*Holopneustes purpurascens*	0	Williamson et al. [33]
*Stypopodium zonale*	*Strongylocentrotus purpuratus*	−	Gerwick and Fenical [31]
**Donor Ochrophyta**	**Target Organism—Arthropoda**	**Effect**	**References**
*Dictyota pfaffii*	*Pachygrapsus transversus*	0	Barbosa et al. [35]
**Donor Ochrophyta**	**Target Organism—Chordata**	**Effect**	**References**
*Dictyopteris delicatula*	*Carassius auratus auratus*	0/−	Alvarez-Hernández et al. [8]
*Dictyota bartayresiana*	*Carassius auratus auratus*	0/−	Alvarez-Hernández et al. [8]
*Dictyota implexa*	*Carassius auratus auratus*	0/−	Alvarez-Hernández et al. [8]
*Dictyota spinulosa*	*Tilapia mossambica*	−	Tanaka and Higa [32]
*Lobophora variegata*	*Carassius auratus auratus*	0/−	Alvarez-Hernández et al. [8]
*Padina gymnospora*	*Carassius auratus auratus*	0/−	Alvarez-Hernández et al. [8]
*Sargassum liebmannii*	*Carassius auratus auratus*	0/−	Alvarez-Hernández et al. [8]
*Stypopodium zonale*	*Carassius auratus auratus*	0/−	Alvarez-Hernández et al. [8]
*Stypopodium zonale*	*Eupomacentrus leucostictus*	−	Gerwick and Fenical [31]
*Stypopodium zonale*	*Eupomacentrus leucostictus*	−	Gerwick et al. [30]

Note: − means inhibiting effects, + means stimulating effect, 0—means lack of effect.

## Data Availability

All data are presented in the article and Supplementary Materials.

## Supplementary Materials

The following are available online at https://www.mdpi.com/article/10.3390/ijms22157865/s1.
