# Supplementary material for: Macroalgal Defense against Competitors and Herbivores
Source: Int J Mol Sci. 2021 Jul 23;22(15):7865. doi: 10.3390/ijms22157865 (PMC8346039; doi:10.3390/ijms22157865)
Supplement: Supplementary file 1 [file ijms-22-07865-s001.zip › ijms-1283576-supplementary.pdf]

**Supplementary material**

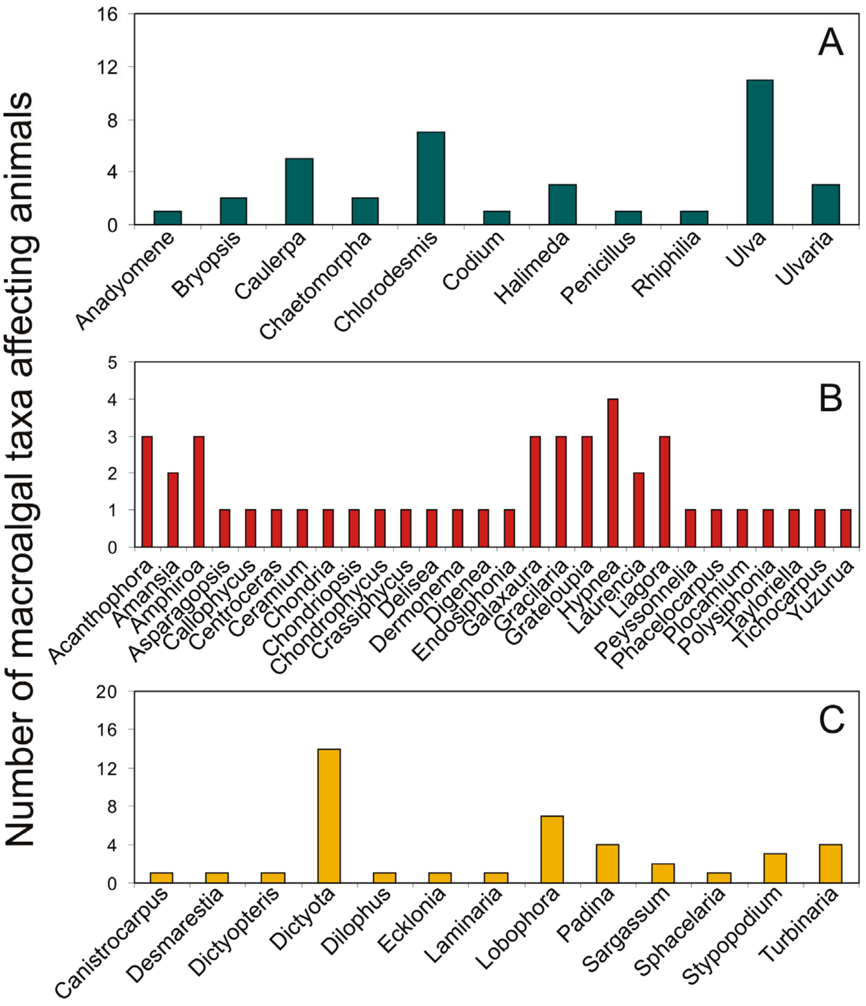


**Figure S1.** Number of allelopathic green algae (A), red algae (B), and brown algae (C) affecting aquatic animals based on taxa found in the literature.

**Table S1.** Studies on donor macroalgae against aquatic organisms published in 32 scientific papers.

| | Article | Place of origin | Macroalgae species | Phylum |
|---|---|---|---|---|
| 1. | Andras et al. (2012) | Votua Reef, Viti Levu, Fiji | *Callophycus densus*<br>*Phacelocarpus neurymenioides*<br>*Plocamium pacificum* | Rhodophyta |
| | | | *Rhiphilia pencilloides* | Chlorophyta |
| 2. | Co Sin et al. (2012) | St John's Island, Singapore | *Bryopsis corymbose*<br>*Halimeda opuntia* | Chlorophyta |
| 3. | Birrell et al., (2008) | Lizard Island on the Australian Great Barrier Reef | *Lobophora variegata*<br>*Padina* sp. | Phaeophyta |
| | | | *Chlorodesmis fastigiata* | Chlorophyta |
| 4. | Bonaldo and Hay (2014) | Coral Coast of Viti Levu, Fiji | *Galaxaura filamentosa* | Rhodophyta |
| | | | *Chlorodesmis fastigiata* | Chlorophyta |
| 5. | Del Monaco et al. (2017) | Heron Island, GBR, Australia | *Chlorodesmis fastigiata* | Chlorophyta |
| | | | *Amansia glomerata* | Rhodophyta |
| | | | *Canistrocarpus* sp. | Phaeophyta |
| 6. | Fong et al. (2019) | Pulau Hantu, Pulau Subar Darat, and Kusu Island, Singapore | *Bryopsis* sp. | Chlorophyta |
| | | | *Endosiphonia horrida*<br>*Hypnea pannosa* | Rhodophyta |

| | | | | |
|---|---|---|---|---|
| | | | *Lobophora* sp. | Phaeophyta |
| 7. | Kuffner et al. (2006) | Long Key, Florida, USA | *Dictyota* sp.<br>*Lobophora variegata* | Phaeophyta |
| | | | *Chondrophycus poiteaui* | Rhodophyta |
| 8. | Longo and Hay (2017) | Moorea, French Polynesia | *Chlorodesmis fastigiata* | Chlorophyta |
| | | | *Amansia rhodantha*<br>*Asparagopsis taxiformis* | Rhodophyta |
| | | | *Dictyota bartayresiana*<br>*Turbinaria ornata* | Phaeophyta |
| 9. | Huggett et al. (2005) | New South Wales, Sydney, Australia | *Ulva australis*<br>*Ulva compressa*<br>*Ulvaria obscura* | Chlorophyta |
| 10. | Morrow et al., (2012) | Belize,<br>Florida Keys, USA | *Halimeda tuna* | Chlorophyta |
| | | Belize | *Dictyota* sp. | Phaeophyta |
| | | Saint Thomas, U.S. Virgin Islands | *Lobophora variegata* | Phaeophyta |
| 11. | Nelson et al. (2003) | Parks Bay, Shaw Island, San Juan County, Washington, USA | *Ulva fenestrata*<br>*Ulva lens* | Chlorophyta |
| 12. | Olsen et al. (2014) | Florida Keys, USA | *Dictyota menstrualis* | Phaeophyta |
| 13. | Paul et al. (2011) | Long Key, Forida, USA | *Dictyota pinnatifida*<br>*Dictyota pulchella* | Phaeophyta |
| 14. | Rasher et al. (2011) | Votua Reef, Viti Levu, Fiji | *Chlorodesmis fastigiata* | Chlorophyta |
| | | | *Amphiroa crassa*<br>*Galaxaura filamentosa*<br>*Liagora* sp. | Rhodophyta |
| | | | *Dictyota bartayresiana*<br>*Padina boryana*<br>*Turbinaria conoides* | Phaeophyta |
| 15. | Ritson-Williams et al. (2016) | Florida Keys, USA | *Chlorodesmis fastigiata* | Chlorophyta |
| | | | *Amphiroa crassa*<br>*Galaxaura filamentosa*<br>*Liagora* sp. | Rhodophyta |
| | | | *Dictyota bartayresiana*<br>*Padina boryana*<br>*Sargassum polycystum*<br>*Turbinaria conoides* | Phaeophyta |
| 16. | Ritson-Williams et al. (2020) | Carrie Bow Cay, Belize | *Dictyota bartayresiana*<br>*Dictyota pulchella*<br>*Lobophora* sp. | Phaeophyta |
| 17. | Tanner (1995) | Heron Island, Great Barrier Reef, Australia | *Chlorodesmis fastigiata*<br>*Halimeda* spp. | Chlorophyta |
| | | | *Peyssonnelia* spp. | Rhodophyta |
| | | | *Turbinaria ornata* | Phaeophyta |
| 18. | Vieira et al. (2016) | New Caledonia | *Lobophora* sp. | Phaeophyta |
| 19. | Webster et al. (2015) | Ningaloo Reef, Australia | *Sphacelaria* sp. | Phaeophyta |
| 20. | Green-Gavrielidis et al. (2018) | Narragansett Bay, Rhode Island, USA | *Ulva compressa*<br>*Ulva lactuca*<br>*Ulva obscura* | Chlorophyta |
| 21. | Nelson and Gregg (2013) | Seattle, Washington, USA | *Ulva lactuca*<br>*Ulvaria obscura* | Chlorophyta |
| 22. | Muñoz et al. (2012) | La Herradura Bay in Coquimbo, Chile | *Ulva* sp. | Chlorophyta |
| 23. | Van Alstyne et al. (2014) | Shannon Point Marine Center, Anacortes, Washington, USA | *Ulvaria obscura* | Chlorophyta |
| 24. | Warkus et al. (2010) | Gulf of Maine | *Chaetomorpha* spp.<br>*Codium fragile*<br>*Enteromorpha* spp.<br>*Ulva lactuca* | Chlorophyta |

| | | | | |
|---|---|---|---|---|
| | | | *Grateloupia turu turu*<br>*Polysiphonia denudata* | Rhodophyta |
| | | | *Desmarestia viridis*<br>*Laminaria* spp. | Phaeophyta |
| | | Dzilam de Bravo, Mexico | *Anadyomene stellata* | |
| | | Mexican Caribbean, the Puerto Morelos reef, Mexico | *Caulerpa cupressoides* | |
| | | Mexican Caribbean, the Puerto Morelos reef, Mexico | *Caulerpa cupressoides* | |
| | | Mexican Caribbean, the Puerto Morelos reef, Mexico | *Caulerpa cupressoides* | Chlorophyta |
| | | Chelem, Mexico | *Caulerpa paspaloides* | |
| | | Faro de Bucerías, Mexico | *Caulerpa racemosa* | |
| | | Faro de Bucerías, Mexico | *Chaetomorpha antennina* | |
| | | Mexican Caribbean, the Puerto Morelos reef, Mexico | *Penicillus capitatus* | |
| | | Costa de Oro, Mexico | *Acanthophora spicifera* | |
| | | Mexican Caribbean, the Puerto Morelos reef, Mexico | *Acanthophora spicifera* | |
| | | Dzilam de Bravo, Mexico | *Acanthophora spicifera* | |
| | | Faro de Bucerías, Mexico | *Amphiroa beauvoisii* | |
| | | La Barrita, Mexico | *Centroceras clavulatum* | |
| | | Mexican Caribbean, the Puerto Morelos reef, Mexico | *Ceramium nitens* | |
| 25. | Alvarez-Hernández et al. (2019) | Costa de Oro, Mexico | *Chondria littoralis* | |
| | | Mexican Caribbean, the Puerto Morelos reef, Mexico | *Chondriopsis dasyphylla* f. *pyrifera* | |
| | | Costa de Oro, Mexico | *Crassiphycus caudatus* (*Gracilaria caudata*) | Rhodophyta |
| | | Puerto Vicente Guerrero, Mexico | *Dermonema virens* | |
| | | Mexican Caribbean, the Puerto Morelos reef, Mexico | *Digenea simplex* | |
| | | Boca del Río, Mexico | *Gracilaria cervicornis* | |
| | | Costa de Oro, Mexico | *Gracilaria cervicornis* | |
| | | Costa de Oro, Mexico | *Gracilaria tikvahiae* | |
| | | Boca del Río, Mexico | *Grateloupia filicina* | |
| | | Alvarado harbour, Mexico | *Grateloupia filicina* | |
| | | Boca del Río, Mexico | *Hypnea musciformis* | |
| | | Costa de Oro, Mexico | *Hypnea musciformis* | |

| | | | | |
|---|---|---|---|---|
| | | La Barrita, Mexico | *Hypnea spinella* | |
| | | Mexican Caribbean, the Puerto Morelos reef, Mexico | *Laurencia obtusa* | |
| | | Champotón, Mexico | *Laurencia obtusa* | |
| | | Mexican Caribbean, the Puerto Morelos reef, Mexico | *Liagora ceranoides* | |
| | | Punta San Telmo, Mexico | *Tayloriella dictyurus* | |
| | | Champotón, Mexico | *Yuzurua poiteaui* var. *gemmifera* | |
| | | Mexican Caribbean, the Puerto Morelos reef, Mexico | *Dictyopteris delicatula* | |
| | | Celestún, Mexico | *Dictyota implexa* | |
| | | Punta San Telmo, Mexico | *Dictyota bartayresiana* | |
| | | Mexican Caribbean, the Puerto Morelos reef, Mexico | *Lobophora variegata* | Phaeophyta |
| | | Faro de Bucerías, Mexico | *Padina gymnospora* | |
| | | Faro de Bucerías, Mexico | *Sargassum liebmannii* | |
| | | Mexican Caribbean, the Puerto Morelos reef, Mexico | *Stypopodium zonale* | |
| 26. | Gerwick et al. (1979) | Carrie Bow Cay, Belize | *Stypopodium zonale* | Phaeophyta |
| 27. | Gerwick et al. (1981) | Carrie Bow Cay, Belize | *Stypopodium zonale* | Phaeophyta |
| 28. | Williamson et al. (2000) | Bare Island, Sydney, Australia | *Delisea pulchra* | Rhodophyta |
| | | | *Ecklonia radiata* | Phaeophyta |
| 29. | Ishii et al. (2004) | Kushiro, Hokkaido, Japan | *Tichocarpus crinitus* | Rhodophyta |
| 30. | Barbosa et al. (2004) | Atol das Rocas, Rio Grande do Norte, Brazil | *Dictyota pfaffi* | Phaeophyta |
| 31. | Suzuki et al. (2002) | Awashima Island, Niigata, Japan | *Dilophus okamurae* | Phaeophyta |
| 32. | Tanaka and Higa (1984) | Kin-cho, Okinawa, Japan | *Dictyota spinulosa* | Phaeophyta |
